# Island Evolution and Systematic Revision of Comoran Snakes: Why and When Subspecies Still Make Sense

**DOI:** 10.1371/journal.pone.0042970

**Published:** 2012-08-24

**Authors:** Oliver Hawlitschek, Zoltán T. Nagy, Frank Glaw

**Affiliations:** 1 Zoologische Staatssammlung München, München, Germany; 2 Joint Experimental Molecular Unit, Royal Belgian Institute of Natural Sciences, Brussels, Belgium; University of California, Berkeley, United States of America

## Abstract

Species delimitation and species concepts have been a matter of debate among biodiversity researchers in the last decades, resulting in integrative taxonomy approaches and the use of modern species concepts, such as the phylogenetic, evolutionary or general lineage species concepts. The discussion of subspecies status and concepts has been addressed much less extensively, with some researchers completely refraining from recognizing subspecies. However, allopatric insular populations that are particularly differentiated have traditionally been assigned subspecies status. We studied the molecular phylogeny and morphology of endemic Comoran tree snakes of the genus *Lycodryas*. Taking an integrative taxonomic approach, we used the concept of independent lines of evidence to discriminate between evidence for specific and subspecific status. Molecular (mtDNA) and morphological data provided sufficient evidence to support four different taxa within Comoran *Lycodryas*. In a revision of this group, we propose two species, each with two subspecies. We present a discussion of the strong sexual dichromatism unique to Comoran *Lycodryas* within the genus and related genera that may be explained by sexual selection in combination with the absence of major predators. Then, we discuss the effects of insular evolution and the “island rule” on morphological traits in Comoran *Lycodryas* and in *Liophidium mayottensis*, another snake endemic to the Comoros. The absence of larger-bodied snakes may have promoted an increase in body size and the number of dorsal scale rows in these species. Finally, we discuss the subspecies concept, its applications and its significance for integrative taxonomy and for limiting taxonomic inflation. We emphasize that taxon descriptions should be based on an integrative approach using several lines of evidence, preferably in combination with statements on the underlying species concepts or operational criteria, to increase the objectivity and comparability of descriptions.

## Introduction

Islands have been regarded as model systems for speciation even by the earliest evolutionary biologists [1], [2]. Particular attention has been given to groups of oceanic islands that have never been connected to other land masses since their (usually volcanic) origins [3]–[10]. In contrast, the Comoros archipelago in the Western Indian Ocean (WIO) has received relatively little attention, possibly because it contains no conspicuous or “odd” endemics, such as the now extinct Dodo (*Raphus cucullatus*) of Mauritius or the Marine Iguana (*Amblyrhynchus cristatus*) of the Galápagos or extensive and characteristic radiations such as drosophilid flies in Hawaii [11] or *Echium* plants in the Canaries [12].

The degree of endemism on the Comoros is not as high as that of comparable island biota although it is considerable: 17 out of 60 species of breeding birds are endemic (28.3%) [13], [14], and Pascal [15] assumes 15% endemism of native plants on the Comoros. The Galápagos have 60.4% endemic birds and 45% endemic plants [16], and the Mascarenes have 66.7% birds and 72% plants [17]. Among terrestrial Comoran reptiles, 13 out of 28 (46.4%) recognized species are endemic, but most of the non-endemics are introduced; if only native species are taken into account, endemism rises to 76.5% according to the current state of taxonomy [18], [19]. All native terrestrial reptiles on the Canaries and the Galápagos are endemic. On Hawaii, there are no native reptile species [20].

For island biota in particular, the question of endemism strongly depends on the underlying taxonomy. A number of both widespread and endemic bird species are present on the Comoros, in addition to island-endemic subspecies. If all these subspecies were to be elevated to species rank, endemism would more than double from 28.3% to 70.0% [14]. In reptiles, the degree of endemism would increase from 46.4% to 60.6% [19]. The degree of endemism, especially of island biota that are generally considered vulnerable [21]–[23], is of high importance in general biodiversity research and conservation planning [24], [25]. It is therefore essential to adopt clear and preferably objective criteria for the delimitation of species and subspecies.

While systematic biology has many categories by which to classify organisms, the rank of species alone is subject to a scientific definition, or rather, ongoing attempts to agree on such a definition. Until the late 20^th^ century, the most widely accepted definition of a species was provided by the biological species concept of Mayr [26] (see also Dobzhansky [27]), who described species as reproductively isolated lineages. Obviously, in most studies on speciation, reproductive isolation was assumed rather than empirically tested. Later, several new definitions of species were proposed based on ecological [28], [29] or phenetic [30] differences between species. Although these definitions would prove useful in many cases, many authors realized that no single set of characters could serve as a universal tool to delimit species. The newly developed species concepts shifted the focus of species delimitation to the questions of recognizing lineages and their degrees of separation. In the evolutionary species concept, species are considered to be independent lineages with own evolutionary tendencies and own historical fates [31]–[33]. Various phylogenetic species concepts exist, sharing the idea that species are clusters of individuals with a parental pattern of ancestry and descent [34]–[37] and a minium diagnosable monophyletic unit [38]. In the general lineage concept, de Queiroz [39] (see also Mayden [33]) defines species as separately evolving metapopulation lineages. These and other [40]–[42] modern species concepts considerably improved our understanding of how to define species. However, testing the proposed criteria in practice and applying them to species delimitations and descriptions has proved to be difficult.

De Queiroz [43] argued that all species concepts established thus far agree that species exist as separately evolving metapopulation lineages, as previously formulated in de Queiroz [39]. In his unified species concept [43], he proposed that the numerous criteria traditionally applied in species delimitation are maintained as “operational criteria”. Thus, lineage separation could be inferred from evidence for intrinsic reproductive isolation, ecological divergence, or differences in molecular genetic characters, among other phenomena. With the advances of molecular techniques in phylogenetics and taxonomy, molecular characters (mtDNA and nDNA) have been increasingly used in species delimitation because they often provide information regarding the degree of lineage sorting, haplotype sharing and hybridization [44]–[47]. Integrative taxonomy [48]–[50] considers these different factors, such as morphological, molecular or ecological ones, to be separate lines of evidence when assigning species status.

Universal definitions, or attempts thereof, do not exist for any taxonomic rank of a higher or lower level than species. Higher ranks are assigned to taxa arbitrarily based on the knowledge and opinion of the taxonomist in question. This practice is also widely adopted for taxa below the rank of species, in which a clear lower limit for recognizing any two entities as distinct does not exist. Therefore, some authors rejected the subspecies category and trinomials already in the mid-20^th^ century [51], [52]. Most species concepts following the biological species concept, including the more theoretical evolutionary, phylogenetic and general lineage species concepts, have not included any recommendations for how to handle subspecies (but see Cracraft [38]). However, various authors proposed subspecies concepts, most of which share the notion that subspecies differ “taxonomically” (by one or more distinctive features) and by their geographical ranges [53]–[55]. Some authors sought to improve this definition. The 75% rule proposed by Amadon [56] states that for a diagnostic character or a set of characters, 75% of the population of a proposed subspecies must lie outside 99% of the range of other populations. According to Böhme's [57] review of the ecological work of Kühnelt [58], geographically separated populations should also show differences in ecological preferences to warrant subspecies status. Despite these proposals, subspecies have never been very popular among invertebrate zoologists [59]. In vertebrate zoology, many herpetologists in particular argued against describing new subspecies or even maintaining existing subspecies [60]–[62] (but see Mulcahy [63]). Most arguments in favor of subspecies were advanced by ornithologists, who argued that the rank of subspecies is helpful in studies of evolutionary divergence and conservation [64], useful for identifying distinct populations within biological species [65], and convenient for managing taxonomic entities that do not warrant species status [66]. Allopatric island forms of species with some degree of morphological variation have been traditionally recognized as subspecies, and in modern herpetology, new subspecies are also described [67]–[70]. Recently, Miralles et al. [71] applied the concept of integrative taxonomy, as discussed in Padial et al. [49], to delimit species and subspecies alike.

Morphological differences between insular taxa and related mainland taxa may result from different selection regimes, from founder effects, or from interactions between these two factors [72], [73]. Most commonly, insular taxa particularly of vertebrates have been recorded as divergent in size, with small animals showing a tendency towards gigantism and large ones towards dwarfism. This phenomenon was observed so commonly that it was termed the “island rule” [28], [74]. While this rule has been confirmed in various studies of mammals [74]–[76], reptiles seem to follow a less clearly directed pattern. Often, both giant and dwarf insular forms of the same taxonomic group are known [77]–[79], suggesting that the mechanisms influencing morphological traits on islands in reptiles warrant further study.

In this paper, we investigate the snakes of the genus *Lycodryas* (Lamprophiidae) in the Comoros archipelago, which have thus far been recognized as one endemic species, *Lycodryas sanctijohannis* Günther, 1879 [80]. These predominantly arboreal and nocturnal snakes are present on all four major islands and several smaller islets and are the only advanced snake species throughout most of their range [19], [81]. They are distinguished among snakes and unique among their congeners in that they display striking sexual dichromatism. In an integrative taxonomic approach, we use morphological and molecular data to revise the taxonomic status of Comoran *Lycodryas*. We discuss the effects of island evolution in *Lycodryas* compared with the second Comoran endemic snake, *Liophidium mayottensis*, and the sexual dimorphism in Comoran *Lycodryas*. Finally, we discuss the designation of the subspecies rank for insular populations.

## Methods

### Sampling

Morphological data and tissue samples from *Lycodryas* and *Liophidium* species were obtained from specimens stored at the Zoologische Staatssammlung München, Germany (ZSM) [19], [82]. To gain additional morphological data on the Comoran taxa, all available historical museum specimens were examined. We use the following abbreviations for zoological collections: Natural History Museum, London, United Kingdom (BMNH); Muséum national d'Histoire naturelle, Paris, France (MNHN); Senckenberg Naturmuseum Frankfurt, Germany (SMF); Zoologisches Forschungsmuseum Alexander Koenig, Bonn, Germany (ZFMK); Museum für Naturkunde Berlin, Germany (ZMB); Zoologische Staatssammlung München, Germany (ZSM). A list of all specimens studied is given in Table S1.

Collection and transport of specimens was conducted with the following permits: (1) Issued by the Direction Générale de l'Environnement, Moroni, Union des Comores: research and export permit (no permit number, 1st March 2000), research permit (02/121/MPE/DGE, 12th April 2002), export permit (02/141/MPE/DGE, 2002), research and export permit (no permit number, 12th March 2008), research permit (CNDRS/08/2010, 22nd January 2010), export permit (CNDRS/030/2010, 5th April 2010). (2) Issued by the Direction de l'Agriculture et de la Forêt, Mayotte, France: research and export permit (no permit number, 23rd February 2000), research and export permit (24/DAF/SEF/2008, 19th March 2008), research and export permit (2010-13/DAF/SEF, 30th March 2010). None of the species concerned are listed on CITES appendices. Permission for import to the EU and Germany is not required.

### Morphology

To detect morphological variation between the Comoran *Lycodryas* populations and differences compared with the Malagasy *Lycodryas* species, we studied six morphometrical characters (and five ratios between these characters), thirteen meristic characters, and the colors of 43 specimens. Snout-vent length and tail length were measured to the nearest millimeter and other morphometric characters to the nearest 0.1 millimeter using a digital steel caliper. Meristic characters were examined visually, if necessary using a binocular microscope. Color was considered a reliable character only if photographs or descriptions of living or freshly dead specimens were available. A list of all characters studied, with abbreviations, is given in Table S1. The sex of specimens was determined via dissection of the tail base and inspection of the reproductive organs, if possible.

To detect significant differences among the *Lycodryas* populations of the four Comoro islands, we used Multivariate Analysis of Variance (MANOVA) in PAST version 1.55 [83]. A MANOVA allows testing for equality of the means among several multivariate samples. Given the overall significance of the MANOVA result, pairwise significance tests with Hotelling's p for every pair of island populations are provided. A Canonical Variates Analysis (CVA) is employed to visualize the results.

We also studied six morphometrical characters (and five ratios between these characters) and ten meristic characters in eleven specimens of *Liophidium mayottensis* for comparison with data from the Malagasy *Liophidium* species. Data on the Malagasy species of *Lycodryas* and *Liophidium* were obtained from Glaw & Vences [84] and Nagy et al. [82].

### Laboratory protocols

DNA was extracted from tissue samples of *Lycodryas* using the standard protocols of the Macherey & Nagel NucleoSpin® 96 Tissue kit. We amplified four mitochondrial markers, 16S rRNA (16S), cytochrome *b* (cyt *b*), cytochrome C oxidase subunit 1 (COI), and NADH dehydrogenase 4 (ND4), and one nuclear locus, the proto-oncogene *mos* (c-mos). To test for nuclear divergence below the species level, we also amplified recombination activation gene 2 (Rag2) and the prolactin receptor (PRLR) for the Comoran samples, but these sequences were not included in the overall dataset. The standard PCR protocol used 25 µl reactions with 1 µl of template DNA and the following steps: initial denaturation for 3 min at 94°C, followed by denaturation with 35 cycles of 30 sec each at 94°C, 30 sec of annealing at 47°C and 60 sec of elongation at 72°C, and a final elongation step of 10 min at 72°C. Primer sequences and modifications of the standard PCR protocol are described in Table S2. Sequencing was conducted using the BigDye ® Terminator v1.1 Cycle Sequencing Kit on ABI 3730 and ABI 3130xl capillary sequencers. Sequence data were deposited in GenBank and are available under accession numbers HE798386 to HE798447.

### Phylogenetic analyses

We analysed a dataset of 16S, cyt *b*, COI, ND4 and c-mos with a total of 3498 basepairs. The dataset contained 22 specimens belonging to 9 currently recognized species of the genus *Lycodryas*. We aligned our data with MAFFT 6 [85], [86]. With respect to the different evolutionary characteristics of our molecular markers, we split our dataset into 10 partitions, treating all codon positions of each protein-coding gene and the 16S gene as separate partitions. To identify appropriate substitution models for each partition, we used jModeltest 0.1.1 [87]. We assessed AIC and BIC results, giving BIC preference over AIC.

We conducted maximum likelihood analyses with 1000 fast bootstrap repeats in raxmlGUI 0.93 [88], [89] and Bayesian analyses in MrBayes 3.1.2 [90] on the CIPRES portal 2.2 [91], with two runs and four chains of 30,000,000 generations (samplefreq = 1,000, 25% burnin). MrBayes runs were checked for convergence and normal distribution in Tracer v1.5 [92]. Finally, we conducted parsimony analyses in TNT 1.1 with 1,000 jackknife (removal 36%) replications [93] (hit best tree 5 times, keep 10,000 trees in memory). Pairwise distances were calculated in MEGA 5.0 [94].

We also constructed haplotype networks of all mtDNA markers for Comoran *Lycodryas* using statistical parsimony [95] with a connection limit of 95% in the software TCS v1.21 [96] and manually constructed haplotype networks of all nuclear markers.

### Integrative taxonomy

Currently, the name *Lycodryas sanctijohannis* Günther, 1879 [80] is applied to all Comoran *Lycodryas*. We explore the distinction of all four island populations of this snake as distinct taxonomic units. Vieites et al. [97] distinguish between Unconfirmed Candidate Species (UCS), Confirmed Candidate Species (CCS) and Deep Conspecific Lineages (DCS). UCS are lineages that can be distinguished by molecular characters but that cannot be confirmed by any other means. A CCS is characterized by a detectable genetic differentiation and distinctiveness in at least one character that mediates a reproductive barrier or is known to be of value for species discrimination in the taxonomic group concerned and/or sympatric occurrence with other lineages without admixture. In contrast, DCL are characterized by the absence or only slight expression of differences in characters that mediate a reproductive barrier or are known to be of value for species discrimination and/or indications of admixture with other species.

After testing these criteria, we follow Miralles et al. [71] (see also [98]) in using three different lines of evidence, based on independent datasets to clarify the taxonomy of Comoran tree snakes and assign specific or subspecific status. Each candidate species may qualify for the following lines of evidence: (1) mtDNA: representation by an independent cyt *b* parsimony network with a connection limit of 95% [99]; (2) nDNA: absence of shared Rag2 haplotypes with any other clade in question [100]; (3) morphology: at least one fixed diagnostic character state (qualitative or significant quantitative) [101]. In congruence with Miralles et al. [71], we apply subspecies status if a candidate species qualifies for only one line of evidence and species status if a candidate species qualifies for two or all lines of evidence. For better comparability, we also use the same molecular markers (cyt *b* and Rag2) as Miralles et al. [71]. After formally applying these criteria, we compare the results with those of other molecular markers and the complete phylogenetic results, we compare the three lines of evidence, and we check for taxonomic plausibility.

### Nomenclatural acts

The electronic version of this document does not represent a published work according to the International Code of Zoological Nomenclature (ICZN), and hence the nomenclatural acts contained in the electronic version are not available under that Code from the electronic edition. Therefore, a separate edition of this document was produced by a method that assures numerous identical and durable copies, and those copies were simultaneously obtainable (from the publication date noted on the first page of this article) for the purpose of providing a public and permanent scientific record, in accordance with Article 8.1 of the Code. The separate print-only edition is available on request from PLoS by sending a request to PLoS ONE, Public Library of Science, 1160 Battery Street, Suite 100, San Francisco, CA 94111, USA along with a check for $10 (to cover printing and postage) payable to “Public Library of Science”.

In addition, this published work and the nomenclatural acts it contains have been registered in ZooBank, the proposed online registration system for the ICZN. The ZooBank LSIDs (Life Science Identifiers) can be resolved and the associated information viewed through any standard web browser by appending the LSID to the prefix “http://zoobank.org/”. The LSID for this publication is: urn:lsid:zoobank.org:pub:F0457CFC-0838-4B81-B063-1435C4D68FD0.

We deposit printed copies of the work in the libraries of the following institutes: Natural History Museum, London, United Kingdom (urn:lsid:biocol.org:col:1004); Muséum national d'Histoire naturelle, Paris, France (urn:lsid:biocol.org:col:34988); Senckenberg Naturmuseum Frankfurt, Germany (urn:lsid:biocol.org:col:34838); Zoologisches Forschungsmuseum Alexander Koenig, Bonn, Germany (urn:lsid:biocol.org:col:34613); Museum für Naturkunde Berlin, Germany (urn:lsid:biocol.org:col:35208); Zoologische Staatssammlung München, Germany (urn:lsid:biocol.org: col:34660).

## Results and Discussion

### Phylogeny

**Figure 1:**
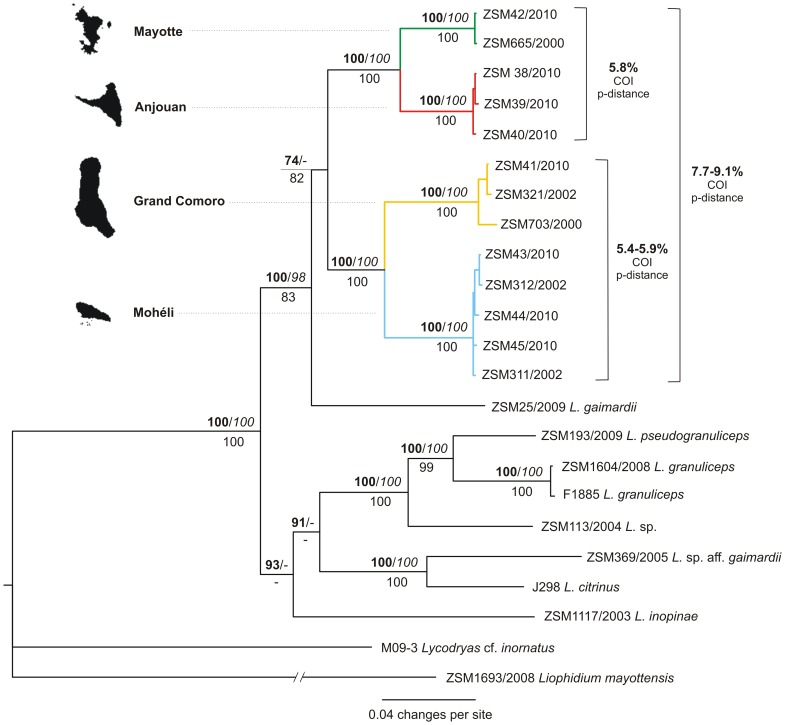
Phylogram of Comoran and Malagasy *Lycodryas*. The tree is based on a Bayesian analysis of a 3498 bp dataset. MrBayes posterior probabilities (*100, bold) and RAxML bootstrap support values (italic) are given above the nodes, TNT jackknife support values are given below the nodes. P-distances of the COI marker, as calculated in MEGA, are given for the entire clade of Comoran *Lycodryas* and for the two pairs of sister taxa. Note that the threshold for species delimitation in lamprophiid snakes, including *Lycodryas*, determined in the barcoding of Malagasy reptiles [104] was 8.3% of COI p-distance.

The tree resulting from our phylogenetic analyses is presented in Fig. 1. The monophyly of all four island populations of Comoran *Lycodryas* is highly supported by all analyses. The sister-group relationships of the Anjouan and Mayotte populations vs. the Grand Comoro and Mohéli populations are equally well supported. All Comoran populations of *Lycodryas* are displayed as a monophyletic group, but with moderate support only. Preliminary analyses with a smaller set of molecular markers presented the taxon *Lycodryas gaimardii*, which had not been included in previous studies, nested within Comoran *Lycodryas*. The multi-gene phylogeny presented in Fig. 1 shows *L. gaimardii* as a sister taxon to all Comoran *Lycodryas*. This view is supported by morphological characters, which clearly distinguish *L. gaimardii* from Comoran *Lycodryas* [82], [102]. Furthermore, the clade including all Comoran *Lycodryas* plus *L. gaimardii* is highly supported. This relationship confirms that *Stenophis* Boulenger, 1896 [103] (type species *Lycodryas gaimardii*) is a junior synonym of *Lycodryas* Günther, 1879 [80] (type species *Lycodryas sanctijohannis*).

**Figure 2:**
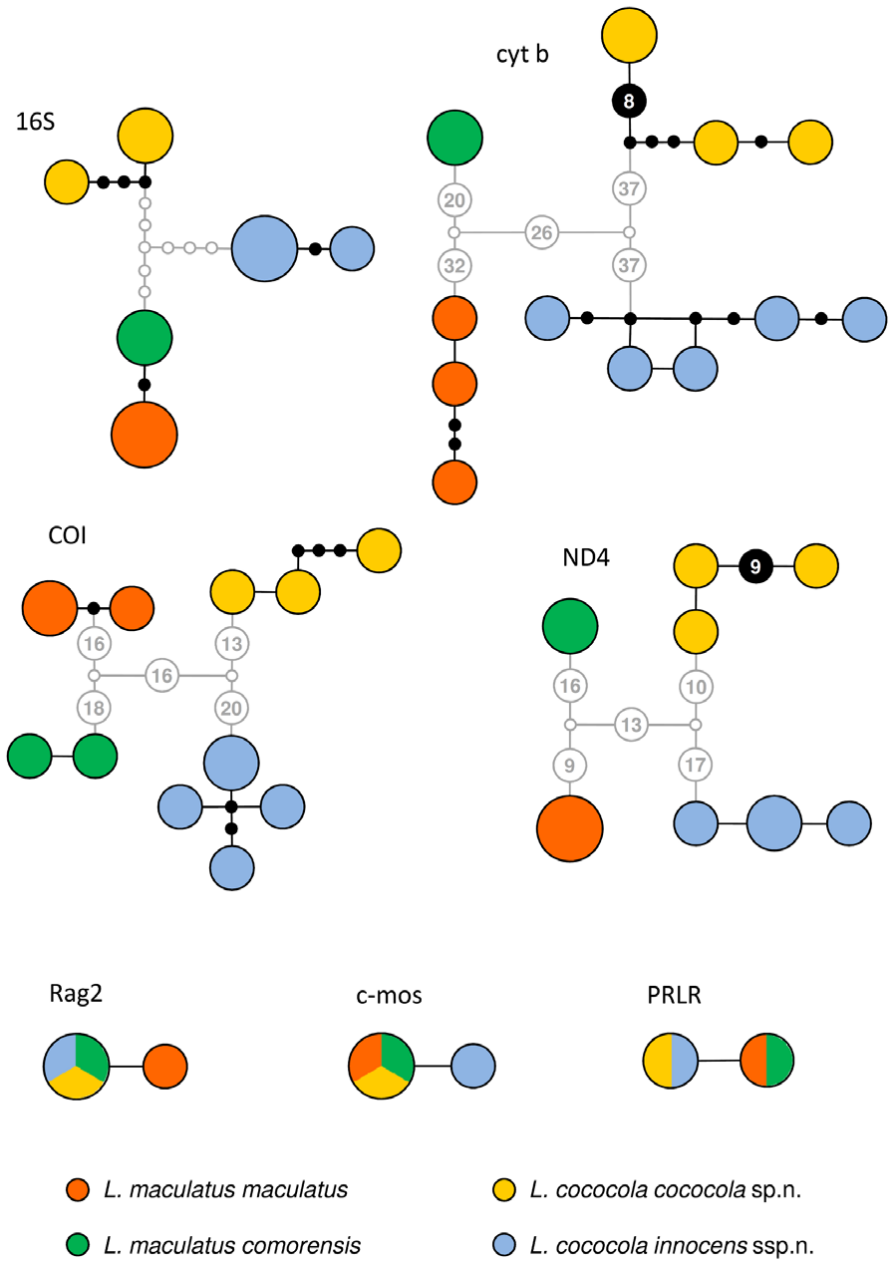
Haplotype networks of all molecular markers analyzed. Colored circles represent haplotypes; larger circles represent haplotypes that are shared by more than one specimen. MtDNA haplotypes that are situated in a common TCS network are connected by black lines, with black dots representing interlying mutation steps. Higher counts of mutation steps are given in numbers. Separate TCS networks are connected by grey lines according to MEGA distance trees. NDNA “networks” were constructed manually.

Under a connection limit of 95%, all four clades of Comoran *Lycodryas* were retrieved as independent parsimony networks in TCS v1.21 for the protein-coding mtDNA markers (Fig. 2). Haplotypes from Mayotte and Anjouan form a common network in 16S. Genetic divergences, measured as uncorrected p-distances of the cytochrome *b* gene, within island populations of Comoran *Lycodryas* are 1.2% or less (maximum in the Grand Comoro population). The divergences between the groups of individuals from Anjouan and Mayotte are 5.3% to 5.5%, between Grand Comoro and Mohéli 7.6% to 7.8%. The distances between these two major Comoran clades are 8.6% to 10.4%. Notably, the distances between samples of *L. granuliceps* and *L. pseudogranuliceps* are 7.1% to 7.2%, and can be as low as 6.1% according to Nagy et al. [82]. These two taxa are poorly differentiated by morphological characters according to Vences et al. [102]. The next shortest distance of 8.5% was measured between *L. citrinus* and *L.* sp. aff. *gaimardii*, which are highly distinct from each other by morphology and coloration. All other interspecific divergences measured within *Lycodryas* were comparable to or higher than the level expressed within Comoran *Lycodryas*.

P-distances in the COI gene are 5.8% between the Anjouan and Mayotte populations, 5.4% to 5.9% between the Grand Comoro and Mohéli populations, and 7.7% to 9.1% between these two clades (Fig. 1). Nagy et al. [104], in a DNA barcoding study of Malagasy reptiles, found thresholds for COI divergences between sister species to be specific for various reptile groups. For lamprophiid snakes, including the genus *Lycodryas*, they found a threshold of 8.3%. A comparison shows that the distance within both major clades of Comoran *Lycodryas* is well below this threshold and thus lower than between other closely related species of *Lycodryas*. The distance between these major clades, however, matches this threshold.

Three nuclear markers were analyzed for possible differentiation between the insular populations of Comoran *Lycodryas*. An inspection of chromatogram data did not indicate the presence of heterozygotes, so separation of alleles was not necessary. A single substitution was detected in each 468 bp of PRLR (Mayotte+Anjouan clade vs. Grand Comoro+Mohéli clade), 626 bp of c-mos (Grand Comoro clade vs. other clades), and 613 bp of Rag2 (Anjouan clade vs. other clades). These data support the clades produced by analyzing the entire molecular dataset.

### Morphological variation within Comoran Lycodryas

Comoran *Lycodryas* show an overall relatively high morphological variability. However, this variation extends throughout Comoran *Lycodryas* and is not suited for distinguishing insular populations. This is in contrast to the relatively large genetic distance described above. MANOVA results for an overall comparison of all Comoran *Lycodryas* are not significant. However, pairwise comparisons of meristic characters show significant differences (p<0.039*) for Anjouan-Mayotte, Anjouan-Mohéli, Grand Comoro-Mayotte and Grand Comoro-Mohéli, but not for Anjouan-Grand Comoro and Mayotte-Mohéli. This contradicts the results of the molecular phylogeny, according to which the populations of Grand Comoro and of Mohéli and those of Anjouan and Mayotte form sister groups.

**Figure 3:**
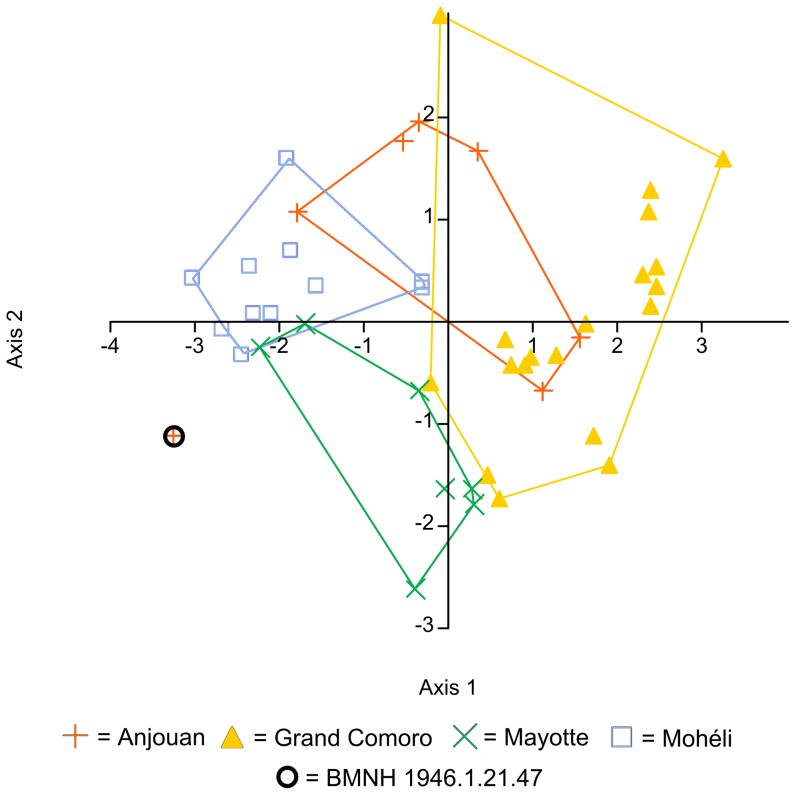
CVA plot of meristic data of Comoran *Lycodryas* species. Characters included are: V (number of ventral scales), MD (number of middorsal scale rows), SC (number of subcaudal scale rows), SLAB (number of supralabial scales – mean of left and right side), ILAB (number of infralabial scales – mean of left and right side), SLCE (number of supralabials in contact with the eye – mean of left and right side). Note that BMNH 1946.1.21.47, the type specimen of *L. maculatus*, represents an outlier of the Anjouan sample due to its uniquely low SLAB and uniquely high SC.

**Figure 4:**
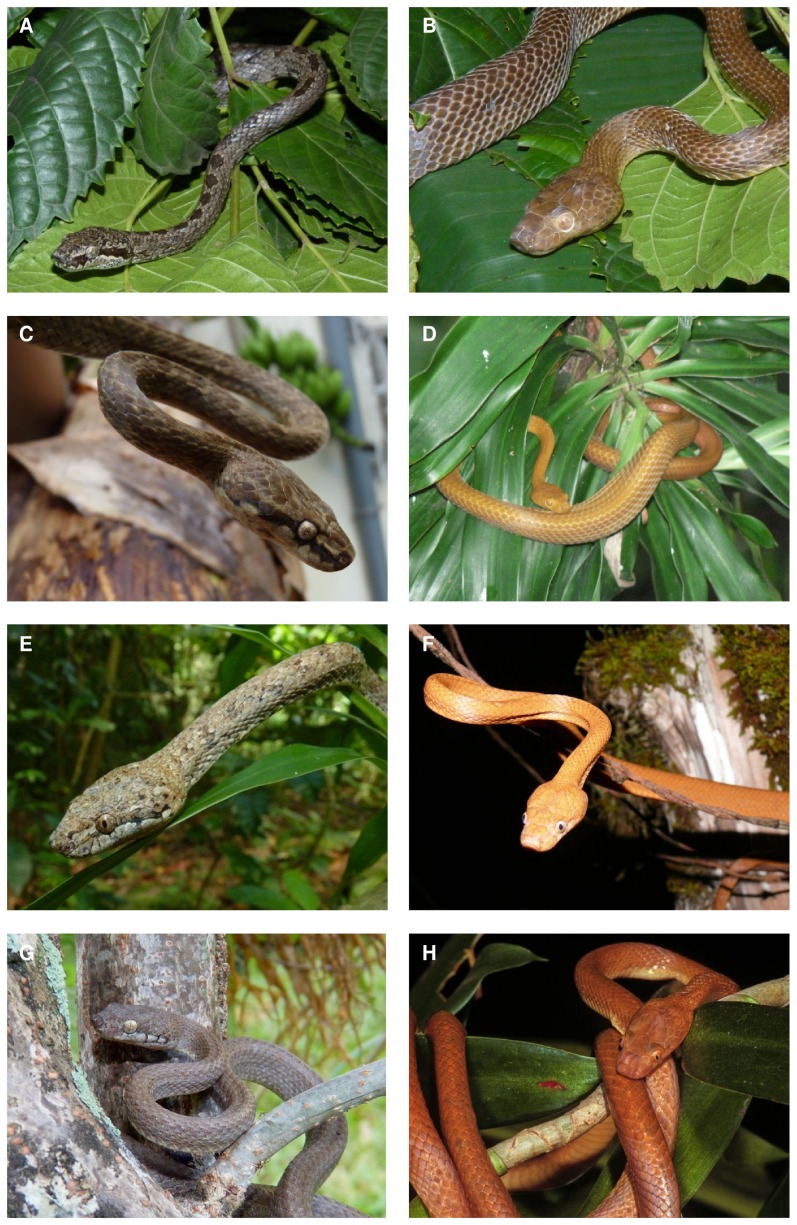
Photographs of Comoran *Lycodryas* specimens. A: ZSM 38/2010, male, Anjouan. B: ZSM 40/2010, female, Anjouan. C: ZSM 42/2010, male, Mayotte. D: female specimen observed at Boungoundranavi, Mayotte. E: ZSM 43/2010, male, Mohéli. F: ZSM 1682/2008, female, Mohéli. G: ZSM 41/2010, male, Grand Comoro. H: ZSM 703/2000, female, Grand Comoro. Photographs A, B, C, E, G by O. Hawlitschek, D by G. Viscardi, F by B. Brenzinger, H by F. Glaw.

The CVA scatterplot (Fig. 3) displayed only partial separation between the insular populations. The characters with the highest loadings on both axes displayed were the number of subcaudal scales (axis 1: −1.84; axis 2: +3.06) and the number of ventral scales (axis 1: +0.85). Table 1 lists these and other characters useful for discriminating between island populations of Comoran *Lycodryas*. Specimens from Grand Comoro are shorter than those from other islands, with a maximum snout-vent length of 650 mm (867 for Anjouan, 757 for Mayotte and 835 for Mohéli). Male specimens from Mayotte are distinguished by a dark ventral line (see also Boettger [105]). The dark dorsal pattern is a character that may be useful in discriminating among males from all islands (Table 1 and Fig. 4), but it appears that this pattern is graded between insular populations. It often fades in preserved specimens. Females on all islands share uniform reddish to yellowish color. Notably, we found specimens on all islands, except for Anjouan, that had only 17 dorsal scale rows at midbody in contrast to the regular 19. No significant morphometrical or meristic differences were detected between sexes in any of the insular populations.

**Table 1:** Morphological data and colors of *Lycodryas* from the four islands of the Comoro archipelago.

	Anjouan (N = 6)	Mayotte (N = 7)	Grand Comoro (N = 19)	Mohéli (N = 11)
SVL	653.83±130.60 (516–867)	602.57±87.60 (504–757)	569.68±80.02 (402–650)	682.64±126.11 (491–835)
MD	19	19 (17)	19 (17)	19 (17)
V	241.83±10.70 (233–259)	238.29±10.92 (227–261)	244.16±7.73 (232–260)	242.18±8.12 (231–255)
SC	95.50±11.78 (85–126*)	95.50±15.59 (84–117)	97.05±9.33 (84–116)	104.11±20.08 (77–140)
SC divided	partly (posterior)	partly (posterior)	partly (posterior)	partly (posterior)
Anal shield	divided	Divided	Divided	divided
SLAB	8–9	8–9	7–10	8
ILAB	8–10**	9	9–11	9–11
SLAB in contact with loreal	2+3	2+3	2	2
Dark ventral line	absent	present (males)	Absent	absent
Male dorsal color	grey with mostly clearly defined dorsal black blotches	grey to olive with pattern of dark spots (sometimes diffuse)	uniformly grey	grey with diffuse pattern of dark spots

SVL = snout-vent length [mm], MD = number of middorsal scale rows at midbody, V = number of ventral scales, SC = number of subcaudal scales, SLAB = number of supralabial scales, ILAB = infralabial scales. In MD, most specimens have 19, but exceptions of 17 were recorded (2 on Grand Comoro, 1 on Mayotte, 2 on Mohéli). No significant morphometrical or meristic differences were detected between sexes on any island.

* : 126 in BMNH 1946.1.21.47, type specimen.

** : 8 only in BMNH 1946.1.21.47.

**Figure 5:**
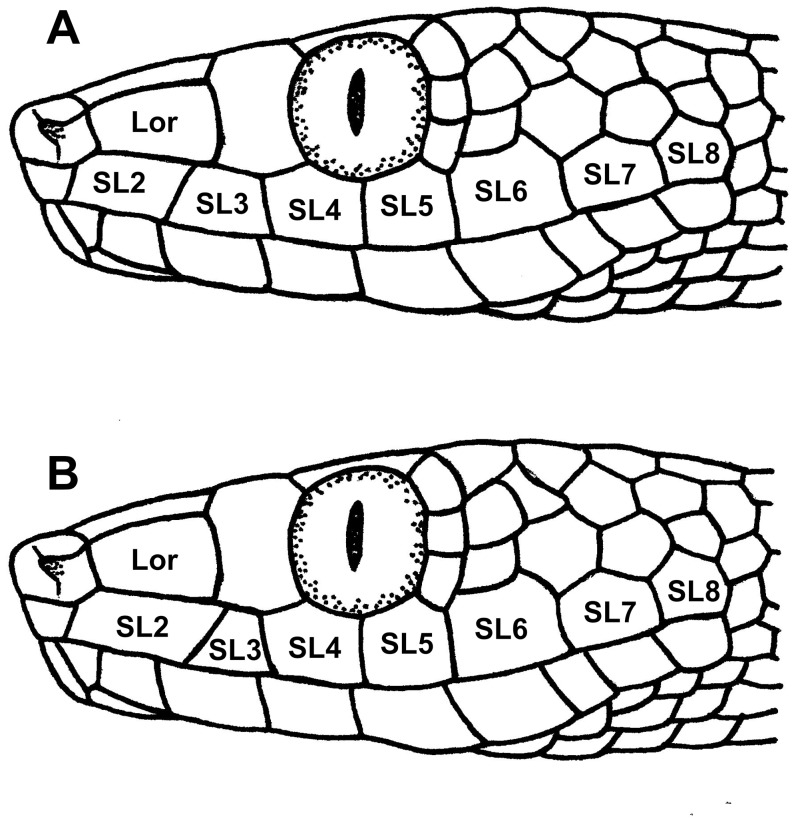
Position of the loreal scale in Comoran *Lycodryas*. Drawings represent specimens from Anjouan and Mayotte (A) or from Grand Comoro and Mohéli (B). In (A), the loreal scale (Lor) is in contact with supralabials (SL) 2 and 3, whereas in (B) it is in contact only with supralabial 2.

One morphological character supports the two sister-clade relationships shown in the phylogram (Anjouan+Mayotte and Grand Comoro+Mohéli). In specimens from Mayotte and Anjouan the loreal scale is in contact with supralabials 2 and 3, whereas in specimens from Grand Comoro and Mohéli it is in contact only with supralabial 2 (Fig. 5).

As already mentioned by Domergue [106], distinguishing sexes by dissection of the tail base and inspection of the hemipenes is difficult in Comoran *Lycodryas* due to well-developed hemiclitores of the females. The hemiclitores of these snakes are very similar in size and aspect to the hemipenes, and the size of both organs seems to vary with reproductive state. Hemipenes have a bifurcate tip, although it is not always easy to identify.

### Taxonomic status of Lycodryas sanctijohannis, discussion of Dipsadoboa maculata and Lycodryas gaimardii comorensis

The taxon *Lycodryas sanctijohannis* was established by Günther [80] and has since been used to refer to Comoran *Lycodryas*. We discuss two further taxa, *Lycodryas gaimardii comorensis* (Peters, 1874) [107] and *Dipsadoboa* (*Lycodryas*/*Stenophis*) *maculata* Günther, 1858 [108], which most likely also refer to Comoran tree snakes.

Peters [107] briefly described a taxon “*Dipsas (Heterurus) gaimardii* Schlegel var. *comorensis*” from Mayotte based on a juvenile specimen that is distinguished from *Lycodryas gaimardii* by a black line over its supralabials, below the eye to the corner of the mouth (often found in *L. sanctijohannis*), and dark crossbands that are narrower than in the typical form. *L. sanctijohannis* often shows patterns of dark blotches and dots but no clearly defined crossbands. It was also described as having 17 dorsal scale rows at midbody, like *L. gaimardii* and unlike *L. sanctijohannis*, which normally has 19. Despite extensive efforts, this specimen could not be traced in ZMB or any other museums that were inspected (R. Günther, M.-O. Rödel, F. Tillack, C. Kucharzewski, pers. comm.). However, the collection at SMF holds a clutch of four hatchlings and three translucent eggs claimed to be collected on Mayotte (SMF 19620 to 19627) and mentioned in Boettger's report on Voeltzkow's travels to the Comoros (in Boettger [105], p. 343) that have been assigned to “*Stenophis* cf. *gaimardi*”. Meirte [81] described *L. sanctijohannis* as ovoviviparous, which may explain the translucence of the eggs. All hatchlings in this clutch have 17 dorsal scale rows and dark dorsal crossbands.

While 19 dorsal scale rows are the rule, we recorded a small number of specimens with 17 dorsal scale rows on all Comoro islands but Anjouan (Table S1). We know of no adult specimens of *L. sanctijohannis* with clear dark crossbands, but no other hatchling or small juvenile specimens were available for examination. Therefore, it is possible that hatchlings (and juveniles) of *L. sanctijohannis* have distinct dark crossbands that become less distinct in adult specimens, similar to other *Lycodryas* species. This leads us to the conclusion that *L. gaimardii comorensis* refers to the *L. sanctijohannis* population from Mayotte.

The taxon *Dipsadoboa maculata* was originally described by Günther (1858, p. 183) based on a single male specimen (BMNH 1946.1.21.47) with “Central America” listed as its type locality. Boulenger [103] (p. 43) placed this taxon in the genus *Stenophis*, provided a redescription and a head drawing and changed the locality to “unknown”. The reasons for rejecting this type locality remain speculative. Günther [107] noted that the specimen was “From M. Parzudaki's Collection”; Parzudaki collected in Central and South America [109]–[111]. Boulenger [103], however, placed the specimen in the genus *Stenophis* based mainly on dental morphology (maxillary teeth “13 or 14, equal”, p. 39–40 vs. “12 or 13, anterior longest”p. 44) and the presence of well-developed hypapophyses throughout the vertebral column. The latter character is used by Boulenger to characterize a very small number of snake genera, including *Stenophis* and *Lycodryas*. Because all other *Stenophis* spp. were from Madagascar, Boulenger most likely doubted the reported origin of the specimen. Notably, the two descriptions of the type specimen disagree in that according to Günther [108], the anal shield is “bifid”, whereas according to Boulenger [103] it is “entire”. Our examination supports the view of Günther.

Nearly all characters in the description of *Dipsadoboa maculata* in Günther [108], as well as in Boulenger [103], fit with *Lycodryas sanctijohannis*. According to C. Kucharzewski (pers. comm., 18 April 2012), the type specimens of *Dipsadoboa maculata* and *Lycodryas sanctijohannis* both have enlarged anterior maxillary teeth and hypapophyses developed throughout the vertebral column and thus show no differences in these characters. In the case of conspecifity, *L. maculatus* (Günther, 1858) [108] would be the oldest and therefore valid name. Consequently, Domergue [106] reported two specimens of *L. maculatus* from the Comoros. The descriptions of the color, pholidosis and morphometrics of these specimens can also be applied to *L. sanctijohannis*. While both Günther [108] and Boulenger [108] state that the holotype has only undivided subcaudal scales, Domergue found that both specimens available to him had anterior undivided and posterior divided subcaudal scales. Our examination showed that the holotype actually has posterior divided subcaudals. Another difference is that both specimens examined by Domergue [106] had “white” supralabials, a common feature of male *L. sanctijohannis* also visible on Boulenger's [103] drawing of this species (plate III), but not on that of *L. maculatus* (plate IV). Our examination of the *L. maculatus* holotype confirmed the absence of white supralabials, but this may be the result of color loss from preservation. Additionally, the holotype has 8/8 infralabial scales, which is less than in all examined specimens of *L. sanctijohannis* in which at least 9/9 infralabials are present.

*L. maculatus* and *L. sanctijohannis* are distinguished from all Malagasy species of *Lycodryas* by having 19 instead of 17 dorsal scale rows. Among related genera of Malagasy tree snakes, the only species exhibiting 19 dorsal scale rows is *Phisalixella iarakaensis* [112]. According to Vences et al. [102], this snake is clearly distinguished from *L. maculatus* by several other characters.

Despite minor differences that may be attributed to intraspecific variation or a poor state of preservation, we conclude that all morphological characters examined suggest conspecifity of *L. maculatus* and *L. sanctijohannis*. We consider the possibility that there is a second species of *Lycodryas* on the Comoros (living in sympatry with the *L. sanctijohannis* complex) to be extremely unlikely, as we could not find any other indication of this despite extensive surveys in the field and of the museum material. The possibility that *L. maculatus* represents a Malagasy species of *Lycodryas* awaiting rediscovery cannot be excluded. Because no such species is known, we tentatively assign the name *maculatus* to Comoran *Lycodryas*. We also cannot entirely exclude that *L. maculatus* actually represents a snake from America or elsewhere in the world, but consider this very unlikely. Examination of the head scalation allows assigning the type specimen to the Anjouan+Mayotte clade, as the loreal scale is in contact with supralabials 2 and 3. Even in poorly preserved male specimens from Mayotte, the dark ventral line was often partly visible, which is not the case in the *L. maculatus* specimen. Therefore, we assign this specimen to the Anjouan population.

*Lycodryas maculatus* (Günther, 1858) [108] is the oldest available name for Comoran tree snakes and thus has nomenclatural priority over *L. sanctijohannis* Günther, 1879 [80]. *Lycodryas gaimardii comorensis* (Peters, 1874) [107] is the oldest available name for the Mayotte population of Comoran tree snakes. We conclude that *Lycodryas maculatus maculatus* (Günther, 1858) [108] is the valid name for Comoran *Lycodryas* from Anjouan and that *Lycodryas maculatus comorensis* (Peters, 1874) [107] is the valid name for *Lycodryas* from Mayotte.

### Lines of evidence for assigning species and subspecies rank to populations of Comoran tree snakes

All four Comoran *Lycodryas* populations are here considered to be candidate species and can be confirmed as distinct taxonomic units (CCS sensu Vieites et al. [97]) according to genetic and morphological data; the status of UCS is not maintained for any clade. The criterion of sympatric occurrence does not apply due to the insular distribution of these clades. We rule out the possibility of DCL because there are no indications of admixture with other lineages.

An inspection of the three lines of evidence (mtDNA, nDNA and morphology) yields the following results: (1) mtDNA: All clades are clearly differentiated by mtDNA markers and are represented by independent cyt *b* parsimony networks. This is confirmed by the parsimony networks based on other markers and by the phylogenetic tree. (2) nDNA: All clades are characterized by an unique combination of nDNA haplotypes. However, the divergence is always limited to a single substitution per marker. Additionally, no single nDNA marker serves to distinguish all four clades. Although all mtDNA markers provide results in accordance with one another, the results provided by the nDNA markers differ from one another, and we cannot exclude the possibility that further study of nDNA markers will yield other results. We therefore assume that these nDNA differences are only slight, not unequivocal and not distinctive enough to be recognized as a line of evidence. (3) Morphology: The position of the loreal scale is a fixed character state distinguishing the Anjouan+Mayotte clades from the Grand Comoro+Mohéli clades. All four clades are distinguished to some degree by differences in male coloration, but these are considered insufficient for completing a line of evidence because they are graded. The smaller size of specimens from the Grand Comoro clade may be a response to ecological constraints rather than a genetically fixed trait.

Strict application of the lines of evidence concept could lead to a view favoring four separate species of Comoran *Lycodryas*: all four candidate species are supported not only by mtDNA but also by differences in nDNA and morphology, albeit only slight differences. In the present case study, we argue that a line of evidence should not be confirmed by such slight differences alone, and we appeal for taxonomic plausibility: Vieites et al. [97] argued that characters to confirm candidate species should be of high value for discriminating species in the respective groups of animals. This is the case for nDNA at a higher level of divergence and for coloration if differences are more significant and possibly quantifiable, but not at levels detected in Comoran *Lycodryas*.

Based on this argument, we resurrect and redescribe *Lycodryas maculatus maculatus* (Günther, 1858) [108] from Anjouan and *Lycodryas maculatus comorensis* (Peters, 1874) [107] from Mayotte. We newly describe *Lycodryas cococola cococola* sp. n. from Grand Comoro and *Lycodryas cococola innocens* sp. n. from Mohéli.

### Redescription of Lycodryas maculatus

Genus *Lycodryas* Günther, 1879 [80]

*Lycodryas maculatus* (Günther, 1858) [108]

Fig. 4: A, B.

http://species-id.net/wiki/Lycodryas_maculatus

#### Original name

*Dipsadoboa maculata* Günther, 1858 [108].

#### Holotype

BMNH 1946.1.21.47; adult male; type locality “Central America”, changed to “unknown” by Boulenger [103].

#### Synonym

*Lycodryas sanctijohannis* Günther, 1879 [80].

#### Holotype

BMNH 1946.1.5.20, adult male; type locality “Anjouan island”.

#### Diagnosis

Largest subspecies of Comoran tree snakes, snout-vent length max. 867 mm; max. snout-vent length for *L. cococola cococola* sp. n. 650 mm, for *L. maculatus comorensis* 757 mm and for *L. cococola innocens* ssp. n. 835 mm. 19 middorsal scale rows, no specimens with 17 known (see Table 1 and Fig. 3 for other taxa of Comoran *Lycodryas*). 233 to 259 ventral scales, 85 to 126 subcaudal scales, posterior ones divided. Loreal in contact with supralabials 2 and 3, as in *L. maculatus comorensis*, but unlike the other two subspecies. Anal shield divided. BMNH 1946.1.21.47 is the only specimen studied with 8/8 infralabial scales, while all other Comoran *Lycodryas* specimens have at least 9/9. Males dorsally grey, head with marbled pattern of darker spots and dots. Lower part of supralabials in males white, upper part brown or black. Body with regular middorsal band of dark brown blotches, scales between these blotches sometimes appearing brighter. Dark ventral stripe always absent. Females with typical pattern of reddish, brownish or yellowish dorsal and yellowish ventral side.

#### Redescription of BMNH 1946.1.21.47 (Holotype of *Lycodryas maculatus*)

Specimen in good condition. No DNA for molecular studies available. Hemipenes not everted. Body slender, approximately as wide as high, snout-vent length 497 mm. Tail complete, length 168 mm. Head clearly distinct from neck, length 14.6 mm. Eye diameter 2.6 mm, pupil vertical, distance between eye and snout-tip 4.2 mm, distance between eyes 2.6 mm.

Scalation: Rostral concave, much wider than tall, hardly visible in dorsal view. Nostrils bordering prenasals, postnasals and supranasals. Loreals each 1 left and right, wider than tall, bordering postnasals, supralabials 2 and 3, preoculars and prefrontals. Supranasals 2, prefrontals 2, frontal 1, preoculars 1/1 (left/right), supraoculars 1/1, postoculars 3/3, parietals 2, supralabials 8/8, 4^th^ and 5^th^ in contact with eye. Mental rhombic, broader than tall. Mental groove separating first infralabials and chin shields, extending to the mental. Infralabials 8/8. Dorsal scales smooth, in 19 rows along the body, ventrals 243, anal shield divided, subcaudals 126, 41^st^ and the posterior 49 divided.

Coloration in preservative. Coloration probably poorly preserved; live coloration unknown. Dorsal and lateral ground color light grayish with irregular brownish shadings, which are probably a result of preservation. Darker grayish or brownish spots smaller than 1 scale distributed over dorsal and lateral sides of body. No regular band of blotches or crossbands visible. The specific name “maculatus” might suggest that distinct spots were originally visible. Head of rather uniformly grayish color; supralabial scales not significantly brighter than rest of head, possibly as a result of poor preservation. Irregular dark blotch extending over the anterior parts of both parietals, bordering frontal, second smaller blotch on posterior part of right parietal. Irregular and asymmetrical shape of blotches suggests they may have resulted from injury. Iris olive-grey. Ventral side of body uniformly brightly grayish without any dark lines or markings.

#### Redescription of BMNH 1946.1.5.20 (Holotype of *Lycodryas sanctijohannis*)

Adult male. Specimen in good condition. No DNA for molecular studies available. Hemipenes not everted. Body slender, approximately as wide as high, snout-vent length 621 mm. Tail complete, length 146 mm. Head clearly distinct from neck, length 16.0 mm. Eye diameter 3.0 mm, pupil vertical, distance between eye and snout-tip 5.5 mm, distance between eyes 5.7 mm.

Scalation: Rostral concave, much wider than tall, hardly visible in dorsal view. Nostrils bordering prenasals, postnasals and supranasals. Loreals each 1 left and right, wider than tall, bordering postnasals, supralabials 2 and 3, preoculars and prefrontals. Supranasals 2, prefrontals 2, frontal 1, preoculars 1/1 (left/right), supraoculars 1/1, postoculars 3/3, parietals 2, supralabials 9/9, 4^th^ and 5^th^ in contact with eye. Mental rhombic to triangular, broader than tall. Mental groove separating first infralabials and chin shields, extending to the mental. Infralabials 10/10. Dorsal scales smooth, in 19 rows along the body, ventrals 259, anal shield divided, subcaudals 85, the posterior 62 divided.

Coloration in preservative. Dorsal and lateral ground color beige to light brownish. Dark brown color elements forming marbled pattern of spots on head and body. Poorly defined row of dark brown blotches visible along vertebral column in anterior third of body, sometimes extending diffusely down the flanks, gradually converting to a diffuse pattern of dark brown spots posteriorly. Flanks with diffuse pattern of smaller dark spots. Supralabial scales beige to whitish, dorsally bordered by dark brown line. Parietals brighter than surrounding scales. Iris olive-brown. Ventral side anteriorly beige with pattern of brown dots, whose density increases posteriorly, turning ventral side almost totally brown towards anal shield (but always lighter grey than dorsal side).

#### Description of ZSM 38/2010

For better comparability with other taxa of Comoran *Lycodryas*, we include the description of an adult male specimen with sequence data and photographs available. Specimen in good condition. Tongue was removed and separately stored as tissue sample for DNA extractions. Hemipenes not everted. Body slender, approximately as wide as high, snout-vent length 688 mm. Tail complete, length 180 mm. Head clearly distinct from neck, length 22.4 mm. Eye diameter 3.3 mm, pupil vertical, distance between eye and snout-tip 6.9 mm, distance between eyes 6.4 mm.

Scalation: Rostral concave, much wider than tall, hardly visible in dorsal view. Nostrils bordering prenasals, postnasals and supranasals. Loreals each 1 left and right, wider than tall, bordering postnasals, supralabials 2 and 3, preoculars and prefrontals. Supranasals 2, prefrontals 2, frontal 1, preoculars 1/1 (left/right), supraoculars 1/1, postoculars 3/3, parietals 2, supralabials 9/8, 4^th^ and 5^th^ in contact with eye. Mental rhombic to triangular, broader than tall. Mental groove separating first infralabials and chin shields, extending to the mental. Infralabials 10/10. Dorsal scales smooth, in 19 rows along the body, ventrals 233, anal shield divided, subcaudals 87, the posterior 60 divided.

Coloration in life. Dorsal and lateral base color grey. Dark brown color elements forming marbled pattern of spots on head. Well-defined row of dark brown blotches visible along vertebral column; scales between these blotches sometimes appearing brighter, especially in anterior body half. Blotches in anterior body half extending diffusely down the flanks, but with sharp contrast between blotches and their lateral extensions. Flanks with diffuse pattern of smaller dark spots. Loreal and prefrontal beige. Lower parts of rostral and supralabials bright beige, similar to ventral color, upper part dark brown, almost black. Frontal and parietals appearing marbled in beige and brown. Iris silvery grey. Ventral side anteriorly beige with pattern of grey dots, whose density increases posteriorly, turning ventral side totally grey towards anal shield (but always lighter grey than dorsal side). Mental with dark median bar connecting to mental groove.

#### Variation

Morphological and chromatic variation is summarized in Table 1 and Fig. 4, respectively.

#### Distribution, natural history and conservation

**Figure 6:**
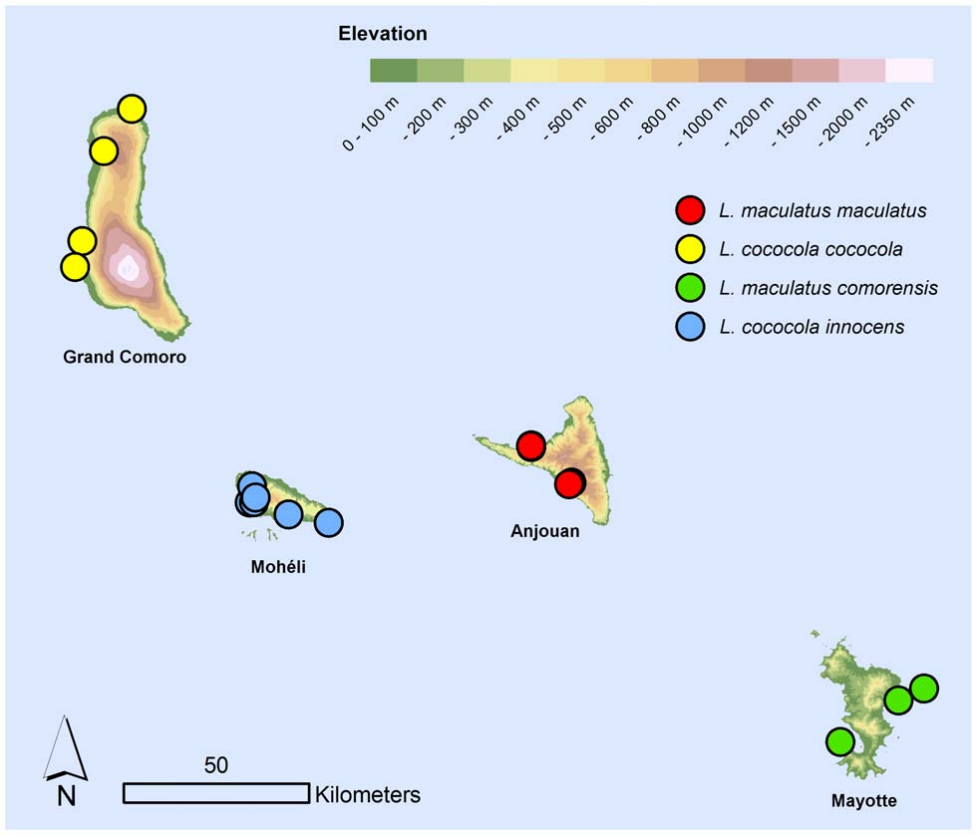
Map of the Comoro archipelago. The circles show records of Comoran specimens of *Lycodryas*.

Endemic to the island of Anjouan. All living specimens for which exact locality data is available were found on trees in plantations and near-natural forests. A distribution map is given in Fig. 6. For geographic coordinates of localities and further comments on habitat and conservation, see Hawlitschek et al. [19].

### Resurrection of *Lycodryas maculatus* comorensis with designation of a neotype

Genus *Lycodryas* Günther, 1879 [80]

*Lycodryas maculatus comorensis* (Peters, 1874) [107]

Fig. 4: C, D.

http://species-id.net/wiki/Lycodryas_maculatus

#### Original name

*Dipsas* (*Heterurus*) *gaimardii* Schlegel var. *comorensis* Peters, 1874 [107].

#### Holotype

Not traced and considered lost, juvenile; type locality “Mayotte island”.

#### Synonym

*Lycodryas sanctijohannis* var. *mayottensis* Boettger, 1913

#### Neotype of Dipsas (Heterurus) Gaimardii Schlegel var. comorensis Peters, 1874

ZSM 42/2010, adult male; collected 7 February 2010; Comoros Archipelago, MAYOTTE, Petite-Terre, near Moya, under bark on a tree; by O. Hawlitschek, J. Berger, B. Brückmann. Justification: The taxon *Dipsas* (*Heterurus*) *gaimardii* Schlegel var. *comorensis* Peters, 1874 [107], has been considered a record of *L. gaimardii* from Mayotte [13]. According to our results, all *Lycodryas* from Mayotte belong to a single taxon that is not conspecific with *L. gaimardii*. Therefore, the designation of a neotype is necessary to stabilize the nomenclatural identity of *Lycodryas* populations from Mayotte.

#### Diagnosis

Subspecies of Comoran *Lycodryas* of intermediate snout-vent length (max. 757 mm); longer than *L. cococola cococola* sp. n. (max. 650 mm), shorter than *L. maculatus maculatus* (max. 867 mm) and *L. cococola innocens* ssp. n. (max. 835 mm). 19 middorsal scale rows, 17 in MNHN 1884-518 (see Table 1 for comparison with other taxa of Comoran *Lycodryas*). 227 to 261 ventral scales, 84 to 117 subcaudal scales, posterior ones divided. Loreal in contact with supralabials 2 and 3, like in *L. maculatus maculatus*, but unlike other taxa of Comoran *Lycodryas*. Anal shield divided. Males can be distinguished from other taxa of Comoran *Lycodryas* by a mostly clearly defined, sometimes diffuse dark stripe extending from the gular region to the tail tip along the row of ventral scales. Dorsal ground color of males grey or olive-grey, the latter being recorded in no other taxa of Comoran *Lycodryas*. Lower part of supralabials in males white, upper part brown or black; pattern of more or less diffuse dark spots extending over the dorsal side. Dark dorsal crossbands were never recorded in any adult specimen. Females show the typical pattern of reddish, brownish or yellowish dorsal and yellowish ventral side.

#### Description of the Neotype

Specimen in good condition. Tongue removed and separately stored as tissue sample for DNA extractions. Hemipenes not everted. Body slender, approximately as wide as high, snout-vent length 757 mm. Tail complete, length 218 mm. Head clearly distinct from neck, length 18.0 mm. Eye diameter 3.8 mm, pupil vertical, distance between eye and snout-tip 7.4 mm, distance between eyes 7.1 mm.

Scalation: Rostral concave, much wider than tall, hardly visible in dorsal view. Nostrils bordering prenasals, postnasals and supranasals. Loreals each 1 left and right, wider than tall, bordering postnasals, supralabials 2 and 3, preoculars and prefrontals. Supranasals 2, prefrontals 2, frontal 1, preoculars 1/1 (left/right), supraoculars 1/1, postoculars 3/3, parietals 2, supralabials 8/8, 3^rd^, 4^th^ and 5^th^ in contact with eye. Mental rhombic, broader than tall. Mental groove separating first infralabials and chin shields, extending to the mental. Infralabials 9/9. Dorsal scales smooth, in 19 rows along the body, ventrals 231, anal shield divided, subcaudals 84, the posterior 36 divided.

Coloration in life. Dorsal and lateral base color olive-grey. Beige, light brown, and dark brown color elements forming diffuse pattern of spots on head and body. , Ill-defined row of brown blotches is visible along the vertebral column in anterior half of body. Blotches extending diffusely down the flanks posterior to head. They may be remnants of dark crossbands, as visible in the hatchlings of SMF 19620 to 19627, and decribed for a juvenile specimen by Peters [107]. On head, prefrontals and supranasals dark, interrupted by beige half-circle connecting to loreals and preoculars, whose upper half is also beige. Lower parts of rostral and supralabials bright beige, similar to ventral color, upper part dark brown, almost black. Frontal and parietals appearing marbled in beige and brown. Iris silvery grey. Ventral side beige, becoming grey posteriorly. Mental with dark median bar connecting to mental groove, then disappearing and reappearing as dark ventral line on first ventral scale. This line, first less than half as broad as the ventral scales, becomes broader and more diffuse towards the tail tip.

#### Variation

Morphological and chromatic variation is summarized in Table 1 and Fig. 4, respectively.

#### Distribution, natural history and conservation

Endemic to Mayotte island and adjacent small islands; recorded on Grande-Terre, Petite-Terre, and Chissioua Mbouzi. All living specimens for which exact locality data is available were found on trees in at least near-natural forests, dry forests or mangrove. However, we know of observations in gardens and plantations. A distribution map is given in Fig. 6. For geographic coordinates of localities and further comments on habitat and conservation, see Hawlitschek et al. [19].

### Description of *Lycodryas cococola* sp. n

Genus *Lycodryas* Günther, 1879 [80]

*Lycodryas cococola* sp. n.

Fig. 4: G, H.

http://species-id.net/wiki/Lycodryas_cococola

urn:lsid:zoobank.org:pub: urn:lsid:zoobank.org:act:70362EFB-B959-439F-9448-6BC3A8B11CEE

#### Holotype

ZSM 41/2010 (field number FGZC 1512); adult male; collected 13 February 2010; Comoros Archipelago, COMOROS, Grand Comoro, Lac Salé; volcanic rocks around lake (11.37375°S; 43.37306°E, 72 m above sea level); by O. Hawlitschek, J. Berger, B. Brückmann.

#### Paratypes

All from Comoros Archipelago, COMOROS, Grand Comoro. ZSM 703/2000; adult female; collected February 2000; exact locality uncertain, said to be from near Chindini; by local collector. ZSM 321/2002; adult female; collected 09 April 2002; Moroni, garden of hotel “La Grillade”; by F. Glaw, M. Hiermeier, M. Kotrba. ZSM 1679/2008; adult male; collected 25 February 2008; plantation near Mbachilé, under stone near mango tree; by O. Hawlitschek, B. Brenzinger. BMNH 1985.338; adult male; near Moroni; by M. Pinchon. MNHN 1890-31; adult male; collected 25 January 1890; by Humblot. MNHN 1899/213; adult female; collected 1899; by Pobeguin. MNHN 1899/214; adult male; collected 1899; by Pobeguin. MNHN 1902-392; adult male; collected 1902. MNHN 1902-393; adult male; collected 1902. MNHN 1902-394; sex undetermined; collected 1902. MNHN 1902-395; sex undetermined; collected 1902. MNHN 1957-732; adult female; collected 1957. MNHN 1961-657; adult female; collected 1961; by Millot. MNHN 1978-2925; adult male; collected 1978; by Domergue, Lt. Plassant. ZFMK 45944; adult female. ZMB 19266; 3 specimens, 2 adult males, 1 adult female; coast; by Voeltzkow.

#### Etymology

Derived from “*Cocos*”, genus name of the coconut palm, and the Latin lexical suffix “-cola”, meaning “inhabiting”. This taxon is commonly found on and near coconut palms, as also reflected by the French vernacular name “serpent des cocotiers” (Coconut palm snake). The species epithet is used as an invariable noun in apposition.

#### Diagnosis

Smallest subspecies of Comoran *Lycodryas*, snout-vent length max. 650 mm; max. snout-vent length for *L. maculatus comorensis* 757 mm, for *L. cococola innocens* ssp. n. 835 mm and for *L. maculatus maculatus* 867 mm. 19 middorsal scale rows, 17 in BMNH 1985.338 and MNHN 1961-657 (see Table 1 for comparison with other taxa of Comoran *Lycodryas*). 232 to 260 ventral scales, 84 to 116 subcaudal scales, posterior ones divided. Loreal in contact with supralabial 2, like in *L. cococola innocens* sp. n., but unlike the other two taxa of Comoran *Lycodryas*. Anal shield divided. Males dorsally almost uniform grey to brownish. Lower part of posterior supralabials in males whitish, 3 anterior supralabials shareing general head coloration. No dark color elements on supralabials as in other taxa of Comoran *Lycodryas*; whitish supralabials may have darker margins. Dark ventral stripe always absent. Females show the typical pattern of reddish, brownish or yellowish dorsal and yellowish ventral side.

#### Description of the Holotype

Specimen in good condition. Tongue removed and separately stored as tissue sample for DNA extractions. Hemipenes not everted. Body slender, approximately as wide as high, snout-vent length 535 mm. Tail complete, length 137 mm. Head clearly distinct from neck, length 18.0 mm. Eye diameter 2.7 mm, pupil vertical, distance between eye and snout-tip 5.1 mm, distance between eyes 5.4 mm.

Scalation: Rostral concave, much wider than tall, hardly visible in dorsal view. Nostrils bordering prenasals, postnasals and supranasals. Loreals each 1 left and right, wider than tall, bordering postnasals, supralabial 2, preoculars and prefrontals. Supranasals 2, prefrontals 2, frontal 1, parietals 2, preoculars 1/1 (left/right), supraoculars 1/1, postoculars 3/3, parietals 2, supralabials 8/8, 3^rd^, 4^th^ and 5^th^ in contact with eye. Mental triangular, broader than tall. Mental groove separating first infralabials and chin shields, extending to mental. Infralabials 10/10. Dorsal scales smooth, in 19 rows along the body, ventrals 233, anal shield divided, subcaudals 87, posterior 77 divided.

Coloration in life. Dorsal and lateral base color of head and body rather uniform grey to brownish. Patterns of darker spots absent. Lower parts of rostral brighter. Anterior 3 supralabials shareing general head color, others whitish, posterior 2 with darker margins. Iris silvery grey. Ventral side yellowish beige without spots or dots. Margins between ventral scales grayish. Upper part of mental darker, no clear stripe visible.

#### Variation

Morphological and chromatic variation is summarized in Table 1 and Fig. 4, respectively.

#### Distribution, natural history and conservation

Endemic to Grand Comoro island. Living specimens for which exact locality data is available were found on trees in plantations and degraded forests, but also on the ground. A distribution map is given in Fig. 6. For additional geographic coordinates of localities and further comments on habitat and conservation, see Hawlitschek et al. [19].

### Description of *Lycodryas cococola innocens* ssp. n

Genus *Lycodryas* Günther, 1879 [80]

*Lycodryas cococola innocens* ssp. n.

Fig. 4: E, F.

http://species-id.net/wiki/Lycodryas_cococola

urn:lsid:zoobank.org:pub: urn:lsid:zoobank.org:act:70362EFB-B959-439F-9448-6BC3A8B11CEE

#### Holotype

ZSM 43/2010 (field number FGZC 1537); adult male; collected 2 March 2010; Comoro Archipelago, COMOROS, Mohéli, near Ouallah (12.32706°S; 43.66918°E, 12 m above sea level); on tree in degraded forest; by O. Hawlitschek, J. Berger, B. Brückmann.

#### Paratypes

All from Comoros archipelago, COMOROS, Mohéli island. ZSM 311/2002; adult female; collected 18 April 2002; East of Nioumachoua; by F. Glaw, M. Hiermeier, M. Kotrba. ZSM 312/2002; adult female; collected 20 April 2002; around Ouallah; by F. Glaw, M. Hiermeier, M. Kotrba. ZSM 43/2010; adult female; collected 2 March 2010; East of Ouallah; on tree in plantation area; by O. Hawlitschek, J. Berger, B. Brückmann. ZSM 45/2010; adult female; collected 30 March 2010; Lac Dziani Boundouni; on tree in degraded forest surrounding lake; by O. Hawlitschek, J. Berger, B. Brückmann. ZSM 1682/2008; adult female, gravid with 3 eggs; collected 5 March 2008; Chalêt St. Antoine; tree in clearing on summit near Chalêt; by O. Hawlitschek, B. Brenzinger. MNHN 8702; adult male; collected June 1957; by J. Millot. ZMB 19227; adult male; by A. Voeltzkow. SMF 19629; 3 specimens, all adult, 1 male, 2 females; collected 1905; by A. Voeltzkow.

#### Etymology

“Innocens”, Latin adjective of identical ending in masculine, feminine and neutral gender, meaning “innocent”. This name was given in reference to the fact that inhabitants of Mohéli are very afraid of this snake and kill many individuals although it is harmless to humans and generally non-aggressive. Although this is also true for other taxa of Comoran *Lycodryas*, local people seem most afraid of snakes on Mohéli.

#### Diagnosis

Large subspecies of Comoran *Lycodryas*, snout-vent length max. 835 mm; max. snout-vent length for *L. cococola cococola* sp. n. 650 mm, for *L. maculatus comorensis* 757 mm and for *L. maculatus maculatus* 867 mm. 19 middorsal scale rows, 17 in ZMB 19227 and 1 specimen of SMF 19629 (see Table 1 for comparison with other taxa of Comoran *Lycodryas*). 233 to 259 ventral scales, 85 to 115 subcaudal scales, posterior ones divided. Loreal in contact with supralabial 2, like in *L. cococola cococola* sp. n., but unlike the other 2 taxa of Comoran *Lycodryas*. Anal shield divided. Males dorsally grey, with pattern of light and darker brown spots. Lower part of posterior supralabials in males white, upper part brown or black; first 2 or 3 supralabials shareing the overall head coloration. In general, the coloration of the supralabials is less apparent that in *L. maculatus comorensis* and in *L. maculatus maculatus*. Dark ventral stripe always absent. Females show the typical pattern of reddish, brownish or yellowish dorsal and yellowish ventral side.

#### Description of the Holotype

Specimen in good condition. Tongue removed and separately stored as tissue sample for DNA extractions. Hemipenes not everted. Body slender, approximately as wide as high, snout-vent length 771 mm. Tail complete, length 200 mm. Head clearly distinct from neck, length 26.2 mm. Eye diameter 3.7 mm, pupil vertical, distance between eye and snout-tip 7.9 mm, distance between eyes 7.5 mm.

Scalation: Rostral concave, much wider than tall, hardly visible in dorsal view. Nostrils bordering prenasals, postnasals and supranasals. Loreals each 1 left and right, wider than tall, bordering postnasals, supralabial 2, preoculars and prefrontals. Supranasals 2, prefrontals 2, frontal 1, preoculars 1/1 (left/right), supraoculars 1/1, postoculars 3/3, parietals 2, supralabials 8/8, 4^th^ and 5^th^ in contact with eye. Mental triangular, broader than tall. Mental groove separating first infralabials and chin shields, extending to mental. Infralabials 10/10. Dorsal scales smooth, in 19 rows along body, ventrals 239, anal shield divided, subcaudals 87, posterior 66 divided.

Coloration in life. Dorsal and lateral base color grey. Diffuse pattern of lighter and darker grey and brown elements dorsally on head, extending onto body, but there with ill-defined small dots and spots of darker brown, also of lighter grey. Lower parts of rostral bright beige, of supralabials darker beige, darker than ventral color, upper part dark brown, almost black. Pattern more contrasted after conservation of specimen, if compared to life photograph. Iris silvery grey. Ventral side yellowish beige with blackish dots arranged in two irregular rows, darkening posteriorly. Mental showing dark median bar not connecting to mental groove.

#### Distribution, natural history and conservation

Endemic to Mohéli island. All living specimens for which exact locality data is available were found on trees in various kinds of forest-like habitats, including plantations, degraded forests and natural forests. A distribution map is given in Fig. 6. For additional geographic coordinates of localities and further comments on habitat and conservation, see Hawlitschek et al. [19].

### Sexual dimorphism

Comoran *Lycodryas* express strong sexual dichromatism (Fig. 4), a very unusual phenomenon among snakes. Differences in morphometry, such as different total length, tail length or head proportions related to sex are observed in a number of snake species, such as Malagasy *Liopholidophis* [84] and many other species across all major families [113]). The expression of different color morphs within populations (e.g., *Vipera berus*, *Madagascarophis colubrinus*), sometimes related to life stage (e.g., *Agkistrodon contortrix*, *Morelia viridis*), is equally common [47], [114]. However, differences in coloration between the sexes are rare and often restricted to slight variations in pattern or shading [84], [113] (and note the possibility of cryptic dichromatism [115]). The color morphs of the Malagasy *Ithycyphus miniatus* have been considered to be related to sexes [116], but this is not supported by more recent observations (pers. obs.). One of the most striking examples of sexual dimorphism in snakes is presented by the Malagasy tree snake *Langaha madagascariensis* in which the sexes differ not only in coloration but also in the form of their rostral appendages [116]. Males are brown dorsally and females are greyish with darker spots, thus displaying a pattern opposite that of Comoran *Lycodryas* in which males are grey with spots and females are brown. Notably, among all species of *Lycodryas* and related genera in the family Lamprophiidae, the Comoran forms alone express this sexual dichromatism [116], [117].

For *Langaha*, Krysko [118] hypothesized that the sexual dimorphism in the form of the rostral appendages may reflect microhabitat differences. This may also apply to color because selection pressures from different types of microhabitats could be assumed to favor different patterns of camouflage. For *Langaha madagascariensis*, this hypothesis seems plausible because this species is at least partly diurnal and has been shown to rely heavily on camouflage for foraging as well as predator avoidance [118]. Unfortunately, sufficient ecological data for testing the microhabitat hypothesis is available neither for *Langaha* nor for Comoran *Lycodryas*. Members of both sexes of the latter have been observed to be active both during the day and night, and the observations do not indicate different microhabitat preferences [19]. Greene [114] (p. 124) speculated that sexual dichromatism in snakes may reflect “divergent antipredator strategies and the increased vulnerability of male snakes as they search for females”. This explanation does not appear to be convincing for Comoran *Lycodryas*. Their sexual dimorphism is expressed in an environment with relatively low predation pressure, given that native mammal and reptile predators are absent [13], and no observations of predation on Comoran *Lycodryas* by birds have been made. Additionally, on Madagascar the intensity of bird predation on snakes seems to be limited [119]–[121] but other *Lycodryas* species showing no sexual dichromatism are preyed upon by various mammals [122]–[123]. This agrees with the results of Macedonia et al. [124], [125], who demonstrated that males of some subspecies of the collared lizard *Crotaphytus collaris* were conspicuously colored in areas with low predator density but cryptically colored in areas with high predator density; conversely, females always exhibited cryptic coloration.

Sexual selection remains a possible explanation for the sexual dichromatism of Comoran *Lycodryas*. In reptiles, this phenomenon has often been observed in lizards [126]–[128] but apparently not in snakes. In Comoran *Lycodryas*, the observation that color patterns of males but not females vary between island populations may be seen as the result of sexual selection by females. In the Malagasy species of *Lycodryas*, including the putative ancestral form of Comoran *Lycodryas*, predation (as discussed above) likely imposed a higher selection pressure on coloration towards camouflage. In contrast, it can be assumed that Comoran *Lycodryas* initially evolved in an insular habitat where predators were scarce. Thus, competition for mates possibly imposed a higher selection pressure on coloration than the need for predator avoidance [124], [125]. Shine [113] argues against the function of color as a sexual signal in snakes because most snakes rely on chemical rather than visual cues to find mates [129], [130], color vision in snakes was poorly developed, and dichromatism rarely takes a form that could be applied in courtship displays (reviewed in Shine [113]). However, later experiments demonstrated that at least some snake species are indeed capable of color perception [131]. The sexual dichromatism in Comoran *Lycodryas* may thus play a role in mate recognition. Even if color perception was poorly developed in this species, the pattern of dark spots on a lighter body expressed by males might function in social signaling based on contrast patterns rather than color. Notably, as mentioned above, the hemiclitores of female Comoran *Lycodryas* are very well developed. Their function is unknown. Females with well-developed hemiclitores are known from the insular pit viper species *Bothrops insularis*, where these specimens that are called “intersexes” by some authors are more numerous than males and (infertile) “normal” females [132]. Reproduction by a female in the absence of males, supposedly through facultative automictic parthenogenesis (FAP), is reported for this species [133]. Possible examples of FAP are recorded in an increasing number of reptiles and certainly provide an advantage for the colonization of new habitats, such as via overseas dispersal [134]. If the founder population of Comoran *Lycodryas* also possessed the ability to reproduce by FAP, then adaptations facilitating mate recognition, such as sexual dichromatism, may have been selected for in order to avoid FAP in favour of sexual reproduction. With the data currently available, however, FAP in Comoran *Lycodryas* remains pure speculation, and additional data should be collected to investigate possible explanations for the phenomenon of sexual dichromatism.

### Effects of island evolution and the “island rule”

Aside from their unique sexual dimorphism, Comoran *Lycodryas* are distinguished from their Malagasy congeners by their larger size. The largest specimens recorded had total lengths of 1052 mm (ZSM 40/2010) and 1047 mm (ZSM 43/2010). The largest Malagasy *Lycodryas* specimen belongs to *L. granuliceps* (formerly *L. capuroni*) and has a total length of 1020 mm [102]. However, most specimens examined by Vences et al. [102] and Nagy et al. [82] were shorter, reaching maximum total lengths of 700 mm or less. In addition to larger body size, Comoran *Lycodryas* have an increased number of 19 dorsal scale rows at midbody, in contrast to 17 in the eight other *Lycodryas* species [82], [102].

The same phenomena are expressed by *Liophidium mayottensis*, the only Comoran endemic in an otherwise Malagasy genus of terrestrial snakes. The maximum total length recorded for this species endemic to Mayotte was 978 mm (Table S1), whereas the largest Malagasy species *L. therezieni* reaches 726 mm according to Glaw & Vences [116]. *L. mayottensis* is also the only species of its genus with 19 dorsal scale rows; all 8 of its congeners have 17 dorsal scale rows [116], [135].

The fact that both arboreal and terrestrial Comoran snakes are larger than their “mainland” congeners (even though Madagascar is an island itself, it can be considered mainland in relation to the much smaller Comoros) conforms to Van Valen's [28] “island rule”. This rule states that on islands, small animals become larger and large animals become smaller in comparison to their mainland relatives. In Van Valen's original work, these phenomena were discussed for mammals alone. Lomolino [74] also discussed it for other vertebrates and described a more general pattern in which island species tend to approach the medium size for their clade (or ‘optimal’ size, see also Boback & Guyer [136]). This again leads to gigantism in small species and dwarfism in larger species (but see Meiri et al. [137]).

Among Malagasy snakes, the genera *Lycodryas* and *Liophidium* represent smaller body sizes in general. Several other genera of terrestrial and diurnal snakes (*Liopholidophis*, *Dromicodryas*, *Leioheterodon*) have larger body sizes than *Liophidium* species, with total lengths of over one meter. The same is true for the arboreal and nocturnal *Lycodryas* and related genera *Parastenophis* and *Phisalixella* [116]. This suggests that on Madagascar, relatively large specimens of *Lycodryas* and *Liophidium* are subject to competition by larger-bodied snake species [116], [138]. Lomolino [74] hypothesized that on islands, where selection pressures due to interspecific competition and environmental heterogeneity are lower, species are less constrained to diverge from their modal size. On the Comoros, each species is the only species representing its guild (terrestrial/diurnal and arboreal/nocturnal snakes, respectively) and interspecific competition if effectively absent. This might have allowed Comoran *Lycodryas* and *Liophidium* to approach their ‘optimal’ body size and become ‘giants’. Notably, the phenomenon of island gigantism is repeated on the Comoros by the iguanid lizard *Oplurus cuvieri comorensis*, a subspecies that attains larger sizes than its mainland congeners: Glaw & Vences [116] state a maximum of 373 mm total length for Malagasy *O. cuvieri* whereas Meirte [81], [139] reports sizes of up to 500 mm for the Comoran subspecies.

The other feature distinguishing both Comoran *Lycodryas* and *Liophidium* from their mainland congeners is their increased number of dorsal scale rows. Previous studies indicated that variation in scale numbers is often correlated with climate; hotter and drier conditions may favor either fewer but larger scales [140], [141] or a greater number of smaller scales [142], [143]. Sanders et al. [144] studied variation in scalation in nocturnal and arboreal *Trimeresurus* snakes, which also show an increase in scale numbers in hotter and drier climates. They argue that larger and often highly sculpted scales are favored if animals are diurnal and exposed to high insulation and thus need to efficiently radiate excess heat. Smaller (and more numerous) scales, however, reduce the area of exposed interstitial skin due to their “tighter fit”. Cutaneous evaporation via the exposed interstitial skin has been shown to be an important way of water loss in reptiles [145]. Since many Malagasy species of *Lycodryas* and *Liophidium* occupy large ranges with variable temperatures and precipitation, it is not easy to establish clear relationships between these factors and their scalation. However, *Lycodryas inornatus* and *L. guentheri* from the dry South and *Lycodryas gaimardii* from the wet east coast of Madagascar both have 17 dorsal scale rows. The same is true for *Liophidium torquatum*, which is from wetter regions all over Madagascar and for *Liophidium apperti*, *L. chabaudi* and *L. trilineatum*, which are from the dry South. The climate on the Comoros is intermediate between these mainland extremes (see climate layers on the worldclim data base [146]). It is therefore an unlikely driving force of variation in scalation.

Instead of climate, the increased number of dorsal scales may simply be correlated with the increase in size and body diameter. Comoran *Lycodryas* have body sizes similar to the larger members of the closely related genus *Phisalixella*, all of which have 19 to 25 dorsal scale rows [82]. These evolutionary trends might also be reflected in the insular species of *Lycodryas* and *Liophidium*.

### The subspecies model

In this paper, we split the formerly recognized single species of Comoran *Lycodryas* into two species, each with two subspecies. However, many authors, particularly herpetologists, have argued against maintaining or newly describing subspecies at all. In the following section, we discuss the subspecies concept in the light of these developments.

As reviewed by de Queiroz [39], [43], different species concepts, or operational criteria of the unified species concept, will identify the split from one into two species at different points in the process of speciation (“considering a separately evolving lineage to have become a species”, according to de Queiroz [43]). Using Cracraft's [38] phylogenetic species concept as an operational criterion, all four island populations of Comoran *Lycodryas* clearly warrant species status: they are monophyletic and diagnosable by differences in the mtDNA markers studied. The monophyletic clusters are supported by the TCS analyses. Thus, they could be regarded as separately evolving metapopulation lineages [39] and may also be seen as independent lineages of the evolutionary species concept [32].

**Figure 7:**
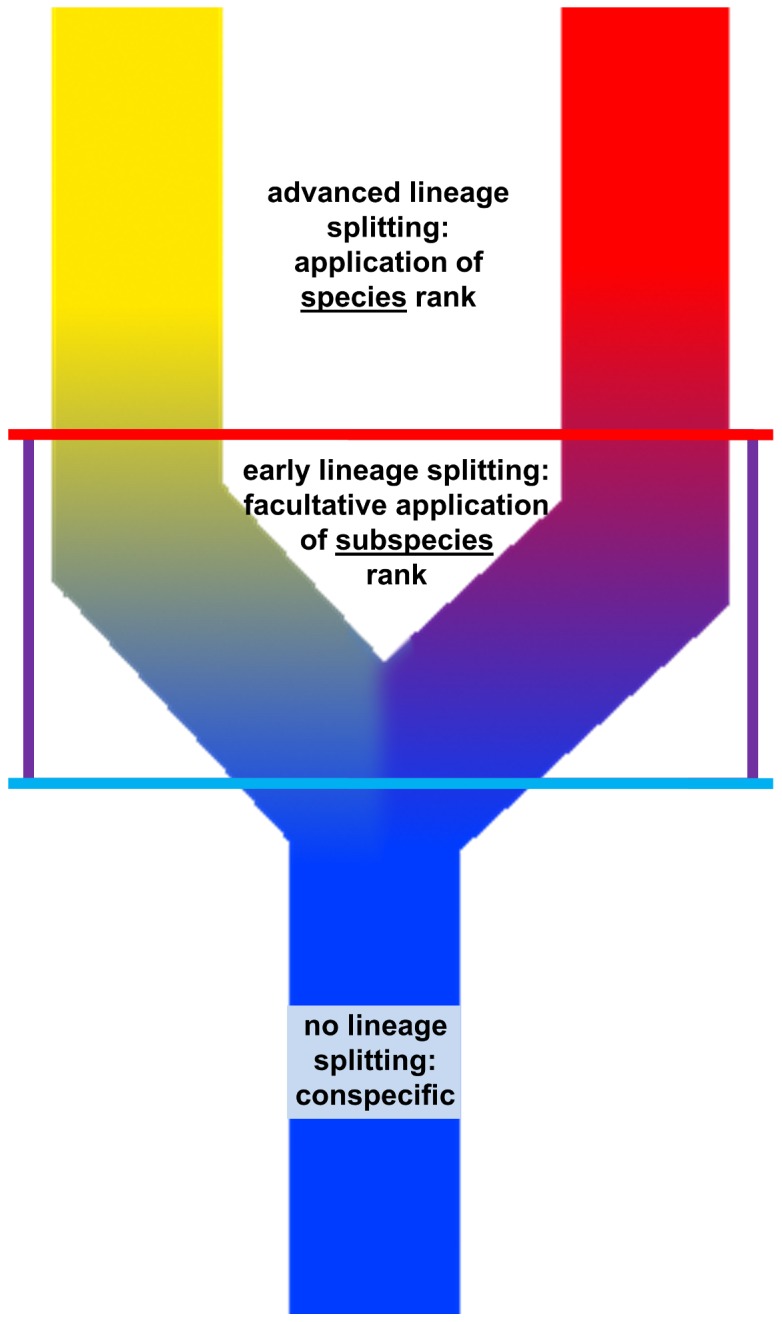
Simplified sketch showing a speciation event. Modified after de Queiroz [42]. A single lineage (blue) splits into two divergent lineages (red and yellow). Below the blue line, a single species is recognized unambiguously, while above the red line, two species are recognized unambiguously. In the zone framed by the lines, disagreement over the number of species (1 vs. 2) is possible, depending on which species concept is applied. Assigning subspecies rank to lineages in this stage appears as a possible solution.

As acknowledged by de Queiroz [43], the question of whether the lineages studied are separate enough to qualify for species status remains. Clearly, all four lineages of Comoran *Lycodryas* share at least similar evolutionary tendencies and a common historical fate [32] due to their insular endemism, origin via overseas dispersal, lack of competition, and similar habitat and ecology. Whether their evolutionary tendencies and their historical fates are just similar or the same remains a decision that can hardly be bolstered by quantitative data and is thus arbitrary. Unfortunately, we feel that the data available for Comoran *Lycodryas* is at a level that allows neither unambiguous acceptance nor rejection of species level for the lineages. The morphological differences are scarce, but morphological diagnosability is not seen as a requirement for most species concepts (explicitly stated in Wiley [32]). A single diagnostic morphological character is found between, but not within, the two major clades of Comoran *Lycodryas* (Anjouan+Mayotte vs. Grand Comoro+Mohéli). The divergence in the nDNA markers studied is marginal, thus rather discouraging elevation to species rank, although it is known that many otherwise well-delimited species show no divergence in nuclear markers [147], [148]. The most significant line of evidence is mtDNA. Divergences are higher between the two major clades confirmed by morphology, and lower within these clades. Notably, as stated above, the divergence between the major clades meets the threshold that was identified for lamprophiid snake species in Nagy et al. [104], while divergences within these clades are lower. Convergently, the divergences in cyt *b* between the major clades are higher than the lowest divergences between other congeneric species, whereas within-clade divergences are in the same range or below. Proposals for thresholds of mtDNA divergence for species delimitation have been made [97], [149], [150], but these are arbitrary and should be used for the preliminary designation of UCS or operational taxonomic units (OTU) rather than for species descriptions, as stated by the authors themselves. Nevertheless, we believe that the comparison with a threshold can be helpful in critical cases, such as that of Comoran *Lycodryas*. Thus, as discussed after the application of the “Lines of Evidence” approach, our data provide relatively good support for splitting Comoran *Lycodryas* into two species, but less convincing support for splitting it into four species. In our view, evidence suggests that speciation within the two major clades has reached a level that does not yet warrant species status. Unlike many authors who decline to recognize a taxonomic level below species [38], [51], [60]–[62], we adopt the view that there is a level of divergence on the way to speciation at which lineages are already diagnosably distinct, but should not yet be considered full species. In our opinion, this level corresponds to the rank of subspecies (Fig. 7). Below, we will provide our reasoning for this view.

Subspecies have largely been neglected in species concepts other than the biological species concept because the main difference between species and subspecies is reproductive isolation [26], [151]: subspecies differ from other subspecies of the same species but can interbreed with them. In the sense of Mayr [26] and Dobzhansky [27], reproductive isolation means isolation by intrinsic mechanisms and not by geographical barriers (as is the case in allopatric island populations). Finding evidence for intrinsic isolation mechanisms has always been a major issue in species delimitation. Therefore, many researchers adopted a view in which species were fully differentiated forms that could coexist in sympatry and allopatric differentiated forms were assigned subspecies status [67], [152], [153]. This corresponds with the definition of subspecies as a “stage in the process of allopatric speciation” [26], [151]. Maintaining reciprocal isolation of parapatric or sympatric species despite the possibility to interbreed is seen as evidence for the existence, and also as reason for the evolution, of intrinsic isolation mechanisms [154].

For these reasons, insular forms with a certain degree of differentiation have often been described as subspecies. In part, this has contributed towards the tendency of herpetologists to reject subspecies. In the Mediterranean region, 23 subspecies of *Podarcis pityusensis* and 91 subspecies of *P. siculus* were recognized at some point. Many of these subspecies descriptions were based on poor evidence, representing local color morphs only and most were later synonymized. Other forms, for which good evidence existed, were elevated to species rank [155]–[159]. Such events have led many authors to reject the subspecies concept as a whole; we argue that this critizism should be directed against such misuse of the subspecies rank, but not to the concept of the subspecies per se. On the Comoros, two examples of island-specific subspecies groups exist. The first example is the four island-endemic subspecies of the skink *Cryptoblepharus boutonii* (Desjardin, 1831) [160], which are now mostly considered species according to Horner [161]. These are easily distinguished by their color patterns but show only shallow genetic divergence and are thus likely to be the result of very recent colonization events [162]. The second example is the radiation of *Phelsuma* day geckos endemic to the Comoros. One island (Mayotte) has two endemic species that most likely resulted from sympatric speciation and are clearly distinct in morphology, ecology and molecular characters. Their sister taxon is *Phelsuma v-nigra* Boettger, 1913 [104], which is present on the remaining three major islands with each one endemic subspecies [163]–[165]. The three subspecies are genetically distinct, show moderate morphological divergence, and have and no determinable differences in ecology [166]. Apparently, the divergence of these three lineages is less strong than between this clade and the Mayotte lineages; therefore, we can see the *P. v-nigra* subspecies as a “stage in the process of allopatric speciation” [26], [151].

If seen as separately evolving metapopulation lineages according to the general lineage concept [39] or minimum diagnosable units according to Cracraft's [32] phylogenetic species concept, all of these geographically isolated insular subspecies would have to be elevated to specfic status. In the case of *Cryptoblepharus boutonii* alone, which inhabits not only the Comoros but a large number of islands in the Indian and Pacific oceans, this would lead to an increase in species numbers from one to 36 [162] (but see Horner [161]). Such an increase in species numbers was discussed broadly in papers on taxonomic inflation [167]–[170]. Taxonomic inflation in species is a problem because species are the taxonomic level that is most important inside and outside taxonomy: it is species, much less subspecies, genera or families, that are counted in species lists, evaluated for conservation purposes and are given most attention in evolutionary studies [64], [65], [171]. Describing minimum diagnosable units as species, or elevating such subspecies to species level, bears the risk that the lower limit of what is diagnosable will be reduced to the point at which diagnosable units no longer represent separately evolving entities. Subspecies, for which neither monophyly nor any type of unique evolutionary tendencies or historical fate are required, are not necessarily concerned by this problem: they describe a level below that of independently evolving lineages [63]. Fitzpatrick [66] considered this level a “zone of art” and subspecies a “heterogeneous mix of evolutionary phenomena”, and before him, Mayr [172] saw subspecies as “convenient handles by which to describe, sort, store, retrieve, and discuss certain types of information about phenotypic geographic variation”. Species, however, are thought to represent real evolutionary entities. We therefore believe that describing species purely based on a personal and subjective interpretation of “existence as a separately evolving metapopulation lineage”, “minimum diagnosable unit” or lineage with “own evolutionary tendencies and historical fate” can lead to very divergent species counts, the same problem for which the subspecies was widely rejected.

While the search for a single overarching species concept continues [173], species delimitations have become more sophisticated in relation to earlier, purely descriptive approaches [174]. We argue that species delimitations should rely on more than one operational criterion and should incorporate modern criteria, such as the evolutionary and the general lineage concept, and guidelines for using the evidence provided by all types of data available. The lines of evidence approach by Miralles et al. [71] provides such a guideline. This concept is arbitrary in and of itself due to its definitions of species and subspecies according to the lines of evidence. Additionally, as shown in the present case study, the lines of evidence should not be followed blindly but instead be judged with a taxonomist's knowledge of related examples, more generalized species concepts and common sense. Nevertheless, the widespread use of integrative taxonomic approaches, with the application of a common guideline, may increase the objectivity of taxon descriptions in all groups of organisms. It may do so more than another new species concept.

## Supporting Information

Table S1 **Morphological data on specimens of** ***Lycodryas*** **from the Comoro islands and** ***Liophidium mayottensis*** **.**
(XLS)

Table S2 **Primers and PCR protocols. Forward (F) and reverse (R) primers are given, all in 5′-3′ order.** Mitochondrial gene loci: 16S = 16S ribosomal RNA, cyt *b* = cytochrome B oxidase, ND4 = NADH dehydrogenase subunit 4, COI = cytochrome C oxidase 1. Nuclear gene loci: c-mos = nuclear genomic proto-oncogene *c-mos*, Rag2 = recombination activating gene 2, PRLR = prolactin receptor.
(PDF)
