# Supplementary material for: Island Evolution and Systematic Revision of Comoran Snakes: Why and When Subspecies Still Make Sense
Source: PLoS One. 2012 Aug 24;7(8):e42970. doi: 10.1371/journal.pone.0042970 (PMC3427315; doi:10.1371/journal.pone.0042970)
Supplement: Table S2 — Primers and PCR protocols. Forward (F) and reverse (R) primers are given, all in 5′-3′ order. Mitochondrial gene loci: 16S = 16S ribosomal RNA, cyt b = cytochrome B oxidase, ND4 = NADH dehydrogenase subunit 4, COI = cytochrome C oxidase 1. Nuclear gene loci: c-mos = nuclear genomic proto-oncogene c-mos, Rag2 = recombination activating gene 2, PRLR = prolactin receptor. (PDF) [file pone.0042970.s002.pdf]

Appendix 2: Primers and PCR protocols. Forward (F) and reverse (R) primers are given, all in 5'-3' order. Mitochondrial gene loci: 16S = 16S ribosomal RNA, CytB = cytochrome B oxidase, ND4 = NADH dehydrogenase subunit 4, COI = cytochrome C oxidase 1. Nuclear gene loci: c-mos = nuclear genomic protooncogene *c-mos*, Rag2 = recombination activating gene 2, PRLR = prolactin receptor.

**Marker:**	**PCR protocol modifications:**	**Primer:**	**Primer Sequence:**	**Reference:**
16S	annealing: 55°C	16sa-l (F)	CGCCTGTTTATCAAAAACAT	Palumbi et al. (1991)
		16sb-h (R)	CCGGTCTGAACTCAGATCACGT	
CytB	40 PCR cycles	L14910 (F)	GACCTGTGATMTGAAAAACCAYCGTTGT	Burbrink et al. (2000), de Queiroz et al. (2002)
		L14919 (F)	AACCACCGTTGTTATTCAACT	
		H16064 (R)	CTTTGGTTTACAAGAACAATGCTTTA	
		L14903 (R)	GACCTGTGATMTGAAAAACCA	
ND4	annealing: 55°C	ND4 (F)	CACCTATGACTACCAAAAGCTCATGTAGAAGC	Arévalo et al. (1994)
		Leu (R)	CATTACTTTTACTTGGATTTGCACCA	
COI	denaturation: 40 sec<br>annealing: 48.5°C	RepCOI-F	TNTTMTCAACNAACCACAAAGA	Nagy et al. (in press)
		RepCOI-R	ACTTCTGGRTGKCCAAARAATCA	
c-mos	40 PCR cycles	S77 (F)	CATGGACTGGGATCAGTTATG	Lawson et al. (2005)
		S78 (R)	CCTTGGGTGTGATTTTCTCACCT	
Rag2	annealing: 60°C, 45 sec	L562 (F)	CCTRADGCCAGATATGGYCATAC	Vidal & Hedges (2005)
		H1306 (R)	GHGAAYTCCTCTGARTCTTC	
PRLR	annealing: 50°C	PRLR_f1 (F)	GACARYGARGACCAGCAACTRATGCC	Townsend et al. (2008)
		PRLR_r3 (R)	GACYTTGTGRACTTCYACRTAATCCAT	

Arévalo, E., Davis, S. K. & Sites, J. W. (1994). Mitochondrial DNA Sequence Divergence and Phylogenetic Relationships among Eight Chromosome Races of the *Sceloporus grammicus* Complex (Phrynosomatidae) in Central Mexico. Systematic Biology 43, 387-418.

Burbrink, F. T., Lawson, R. & Slowinski, J. B. (2000). Mitochondrial DNA phylogeography of the polytypic North American rat snake (*Elaphe obsoleta*): a critique of the subspecies concept. *Evolution* 54, 2107-2118.

de Queiroz, A., Lawson, R. & Lemos-Espinal, J. A. (2002). Phylogenetic relationships of North American garter snakes: how much DNA is enough? *Molecular Phylogenetics and Evolution* 22, 315-329.

Lawson, R., Slowinski, J. B., Crother, B. I. & Burbrink, F. T. (2005). Phylogeny of the Colubroidea (Serpentes): new evidence from mitochondrial and nuclear genes. *Molecular Phylogenetics and Evolution* 37, 581-601.

Nagy, Z. T., Sonet, G., Glaw, F. & Vences, M. (in press). First Large-scale DNA Barcoding Assessment of Reptiles in a Biodiversity Hotspot. PLoS ONE.

Palumbi, S. R., Martin, A., Romano, S., McMillan, W. O., Stice, L. & Grabowski, G. (1991). The Simple Fool's Guide to PCR, Version 2.0. Privately published, Univ. Hawaii.

Townsend, T. M., Alegre, R. E., Kelley, S. T., Wiens, J.J. & Reeder, T. W. (2008). Rapid development of multiple nuclear loci for phylogenetic analysis using genomic resources: An example from squamate reptiles. *Molecular Phylogenetics and Evolution* 47, 129-142.

Vidal, N. & Hedges, S. B. (2005). The phylogeny of squamate reptiles (lizards, snakes, and amphisbaenians) inferred from nine nuclear protein-coding genes. *Comptes Rendus Biologies* 328, 1000-1008.
